# Trends in overweight and obesity by socioeconomic status in Year 6 school children, Australian Capital Territory, 2006–2018

**DOI:** 10.1186/s12889-019-7645-9

**Published:** 2019-11-12

**Authors:** Zongjian Yang, Hai Phung, Ann-Maree Hughes, Sommer Sherwood, Emily Harper, Paul Kelly

**Affiliations:** 1 Preventive and Population Health, ACT Health Directorate, 2-6 Bowes Street, Phillip, Canberra, ACT 2606 Australia; 2 School of Medicine, Griffith University, Brisbane, Australia; 3 Medical School, Australian National University, Canberra, Australia

**Keywords:** Overweight, Obesity, Socioeconomic status, Children, Physical activity, Sedentary behaviour, Sugar-sweetened drinks

## Abstract

**Background:**

Due to the high prevalence and adverse consequences, overweight and obesity in children continues to be a major public health concern worldwide. Socioeconomic background and health-related behaviours (such as diet, physical activity and sedentary behaviors) are important factors associated with weight status in children. Using a series of height and weight assessments from the Australian Capital Territory Physical Activity and Nutrition Survey (ACTPANS), trends in prevalence of overweight and obesity by socioeconomic status were examined in ACT Year 6 school children between 2006 and 2018.

**Methods:**

The ACTPANS has been conducted every 3 years since 2006. A total of 6729 children were surveyed. Complete data on height and weight were available for 6384 (94.9%) participants. Trends in the prevalence of overweight and obesity and associations between weight status and risk factors (such as socioeconomic status, physical activity, screen time and consumption of sugar-sweetened soft drinks (SSD)) were examined using logistic regression.

**Results:**

The prevalence of overweight and obesity remained stable in girls (from 22.5% in 2006 to 21.6% in 2018) but declined in boys (from 27.8 to 17.9%). During the same period, levels of physical activity increased slightly, while screen time and the consumption of fast food and SSD decreased. Socioeconomic gradient, based on the school-level Index of Community Socio-Educational Advantage (ICSEA), was highly associated with prevalence of overweight and obesity. Since 2006, the estimated prevalence of overweight and obesity has remained high in the lowest SES groups, but a concurrent downward trend was observed in the highest SES group, leading to increasing disparity between SES groups. Children in the lowest ICSEA quintile were more likely to be overweight or obese compared to those in the moderate and highest ICSEA quintiles. Children in lower ICSEA quintiles also reported lower levels of physical activity, higher levels of screen time, and higher levels of fast food and SSD consumption compared to those in higher ICSEA quintiles.

**Conclusions:**

While recent trends in overweight and obesity in ACT children are encouraging, the prevalence remains unacceptably high, especially in those from low socioeconomic backgrounds. Additional prevention efforts are required to address the socioeconomic disparity.

## Background

Overweight and obesity in children continues to be a major public health concern worldwide [1, 2]. Overweight children and adolescents are likely to become overweight adults [3, 4]. Excess weight gain during childhood and adolescence is associated with increased risk of numerous noncommunicable diseases (such as cardiovascular diseases, type 2 diabetes and some cancers) throughout the life-course, resulting in a significant economic burden on healthcare systems [2, 5–8]. In addition to the immediate and long-term adverse health consequences, childhood overweight and obesity also has negative impacts on children’s self-esteem, confidence and academic performance [9, 10].

The prevalence of childhood overweight and obesity has increased substantially since the 1970s and has reached an alarming level in many countries [1, 11]. In 2017–18, almost one in four (25%) Australian children and adolescents aged 5–17 were overweight or obese [12]. Consumption of sugar-sweetened beverages and fast food, inadequate physical activity, and excessive screen time have been identified as risk factors for excess weight gain in children [13]. In developed countries, socioeconomic disadvantage in childhood has been shown to be associated with increased risk of overweight and obesity [14–18]. The mechanisms through which socioeconomic background influences weight status in children are still unclear [15].

Given the significant public health implications, it is essential to monitor the weight status of children and associated risk factors. Although some recent reports have shown that, in Australia and other developed countries, the weight status of some age groups of children and adolescents are stabilising [1, 2, 19–22], other studies suggest that the trend may vary by socioeconomic status (SES) [13, 23, 24]. Public health policies and intervention programs designed to change the obesogenic environment and promote lifestyle change in children may have different outcomes across socioeconomic subgroups [25].

Using data from a triennial survey of Year 6 students, the aim of the current study was to examine the trends in prevalence of overweight and obesity among children in the Australian Capital Territory (ACT) between 2006 and 2018. Differences in prevalence and trends across sex and socioeconomic groups, as well as behavioural factors associated with excess weight gain in children (such as physical activity, sedentary behaviour, and consumption of SSD and fast food), are investigated. Few studies of recent trends in weight status of children by socioeconomic status have been reported in Australian populations [23, 26]. A better understanding of the socioeconomic and behavioural factors associated with excess weight gain in children will enable more effective prevention strategies to be developed.

## Methods

### Participants

Data from a triennial surveillance study of a representative sample of Australian Capital Territory (ACT) Year 6 students, undertaken between 2006 and 2018, were used. The ACT is in the south-east of Australia and contains Canberra, the capital city of Australia. Apart from a change in 2015 from pen and paper questionnaires to tablets, the survey methods were the same in all years.

A single-stage cluster sampling design was used. To ensure proportional representation, schools were first stratified by education sector (i.e. government and non-government) and then a sample of schools were randomly selected in proportion to the ratio of Year 6 children for each school sector. Principals of selected schools were contacted to obtain permission to conduct the survey. If a principal declined to participate, a school from the same school sector (which was selected and kept in reserve at the same time as the main sample) was approached as a replacement school. All Year 6 children of selected schools were invited to take part in the study. The selected schools distributed a letter to parents to inform them of the study and seek permission for their child to participate. Only parents who declined their child’s participation were required to respond (i.e. opt-out consent). Students could also choose not to participate at any stage throughout the study. The overall participation rate ranged from 85.5% in 2006 to 88.5% in 2018. A total of 6729 children were surveyed in 2006, 2009, 2012, 2015 and 2018; of these, 98.6% were aged 11–12 years (Table 1). The study was approved by the ACT Health Human Research Ethics Committee.

**Table 1:** Characteristics of the survey participants

Characteristics	Number (unweighted proportion %)					
	Total	2006	2009	2012	2015	2018
Sex						
Boys	3390 (50.4)	577 (49.2)	666 (48.5)	645 (48.4)	753 (55.6)	749 (50.2)
Girls	3333 (49.6)	596 (50.8	708 (51.5)	687 (51.6)	600 (44.4)	742 (49.8)
Age						
11	4843 (72.0)	827 (70.3)	1003 (73.0)	963 (72.1)	980 (72.4)	1070 (71.8)
12	1794 (26.6)	327 (27.8)	355 (25.8)	355 (26.6)	351 (25.9)	406 (27.2)
10 or 13	92 (1.4)	22 (1.9)	16 (1.2)	17 (1.3)	22 (1.7)	15 (1.0)
Indigenous Status						
Indigenous	250 (3.7)	43 (3.7)	56 (4.1)	38 (2.9)	53 (3.9)	60 (4.0)
Non-Indigenous	6479 (96.3)	1133 (96.3)	1318 (95.9)	1297 (97.1)	1300 (96.1)	1431 (96.0)
School Sector						
Government	3905 (58.0)	764 (65.0)	904 (65.8)	769 (57.6)	759 (56.1)	709 (47.6)
Non-government	2823 (42.0)	411 (35.0)	470 (34.2)	566 (42.4)	594 (43.9)	782 (52.4)

### Data collection

In each survey year, trained survey staff administered the questionnaire to students and conducted height and weight measurements on the school premises. In addition to basic demographic data, such as sex, date of birth and Indigenous status, information about children’s diet, physical activity and sedentary behaviours was collected in the survey questionnaire.

### Weight measurement

Body weight was measured to the nearest 0.1 kg using calibrated digital scales without shoes or heavy clothing. Height was measured to the nearest 0.1 cm using a stadiometer with full extended knees and shoes off. Body mass index (BMI) was calculated by dividing the weight in kilograms by squared height in metres, and then weight status of children were categorised as underweight, normal weight, overweight or obese according to the international cut-offs for children developed by the International Obesity Taskforce for age and sex that correspond to the adult cut-offs of 25 for overweight and 30 for obesity [27].

### Physical activity and sedentary behaviour

Physical activity was measured by the question: ‘Over the past seven days (or a typical week), on how many days were you physically active for a total of at least 60 minutes per day?’ For sedentary behaviour, the questions were: ‘About how many hours a day on weekdays (or weekends) do you usually watch television (including videos and DVDs) in your free time?’ and ‘About how many hours a day on weekdays (or weekends) do you usually use a computer (for playing games, emailing, chatting or surfing the internet, excluding school related work) in your free time?’

### Sugar-sweetened soft drink (SSD) and fast food consumption

Frequency of SSD and fast food consumption were measured by the questions: ‘How often do you usually drink soft drink or other sugar-sweetened soft drinks (e.g. Coke, Pepsi, lemonade and cordial)?’ and ‘How often do you eat food from a fast food outlet (e.g. McDonalds, KFC, pizza and Hungry Jacks)?’ For both, the response options were: never, less than once a week, about 1–3 times a week, about 4–6 times a week and every day. The Australian standard definition of sugar-sweetened beverages (SSB) includes sugar-sweetened soft drinks and cordials, fruit drinks, vitamin waters, energy and sports drinks [28]. However, for this paper, we restricted our definition to sugar-sweetened soft drinks and cordials and chose to use the abbreviation SSD because the questions about consumption of fruit drinks, vitamin waters, energy and sports drinks were only included in the 2015 and 2018 surveys.

### Socioeconomic status

For socioeconomic status, the Index of Community Socio-Educational Advantage (ICSEA) was used as an approximation. ICSEA is an aggregate measure at the school-level indicating the scale of socio-educational advantage of all students attending a school [29]. The calculation of ICSEA is based on information relating to parents’ occupation and education, school geographical location and the proportion of Indigenous students [29]. A lower school ICSEA value indicates a lower level of educational advantage, on average, for students attending the school. Quintiles were calculated based on the distribution of ICSEA scores, ranging from the 20% least advantaged (quintile 1) to the 20% most advantaged (quintile 5).

### Statistical analysis

Descriptive analyses were conducted to characterise the survey sample and distribution of risk factors. Post-stratification sampling weights were calculated to match the population benchmark of ACT Year 6 children by school sector, age and sex. Trends in prevalence of overweight and obesity were examined using logistic regression, taking into account the sampling design and weighting. Statistical significance was determined at *p* value < 0.05 and 95% confidence intervals (CI) for estimated proportions were reported.

Logistic regression was used to assess associations between risk factors and weight status (overweight and obesity), adjusting for sex, Indigenous status, ICSEA quintiles and selected behavioural factors. Because ICSEA is measured at the school level, multilevel model approach was used. Based on the significance of individual predictors (*p* < 0.05) and the goodness of fit of the model (Akaike information criterion values), a stepwise regression method was used for selecting variables for inclusion in the multiple regression model. Adjusted prevalence ratios were derived from the fitted logistic regression model [30]. All statistical analyses were conducted using Stata 15.1 (StataCorp, College Station, TX, USA).

## Results

A total of 6729 Year 6 school children were surveyed between 2006 and 2018. Complete data on height and weight measurements were available for 6384 (94.9%) participants; of these, 1139 (17.8%) were classified as overweight and 303 (4.8%) as obese. Table 1 summarises the characteristics of the study population.

**Fig. 1:**
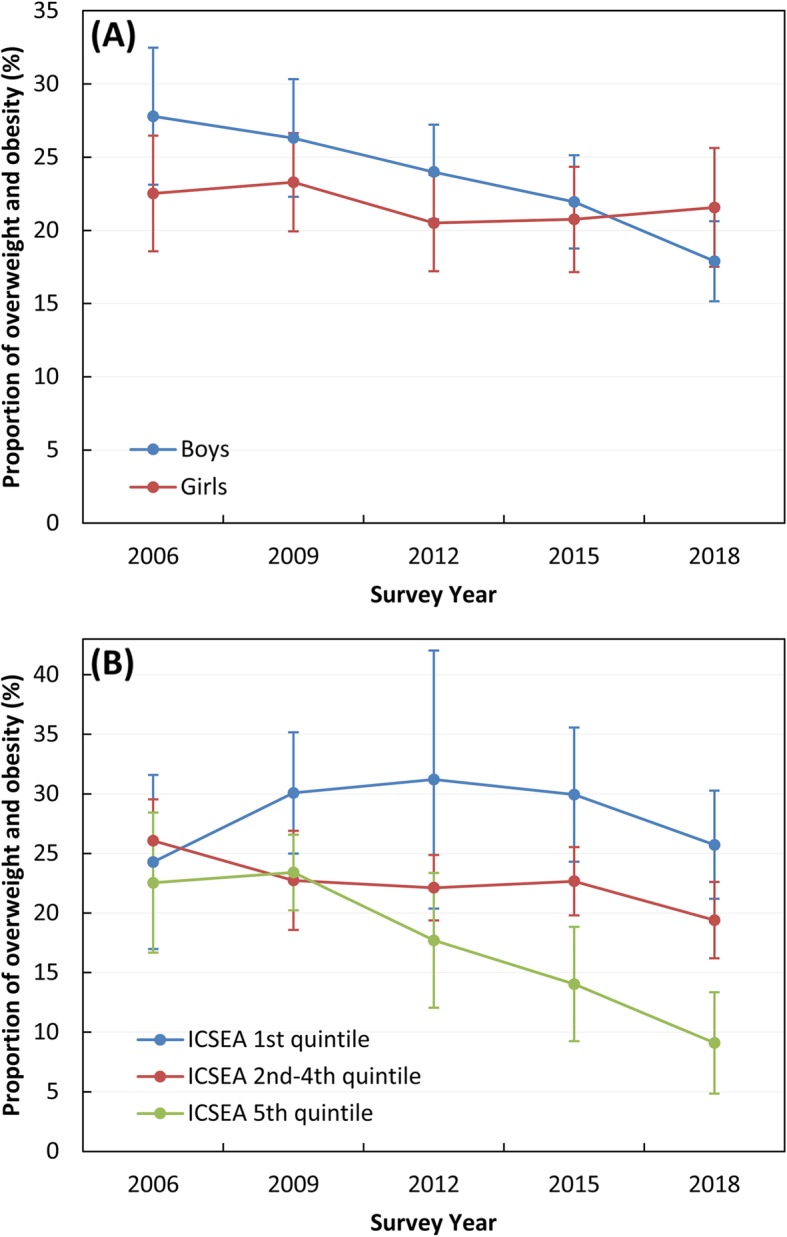
Prevalence of overweight and obesity in ACT Year 6 children, 2006–2018 by sex (**a**) and by quintiles of Index of Community Socio-Educational Advantage (ICSEA) (**b**). Error bars indicate 95% confidence intervals for weighted proportions

Over the 12-year period, the estimated prevalence of overweight and obesity remained stable for girls, from 22.5% (95% CI: 18.6–27.0%) in 2006 to 21.6% (CI: 17.5–26.3%) in 2018 (Fig. 1a). For boys, however, prevalence of overweight and obesity significantly declined from 27.8% (CI: 23.1–33.0%) in 2006 to 17.9% (CI: 15.2–21.0%) in 2018 (Fig. 1a).

Trend analyses by ICSEA showed that the gap between the highest and the lowest socio-economic groups has widened in recent years (Fig. 1b). From 2009 onwards, prevalence of overweight and obesity remained highest in the lowest ICSEA quintile, whereas a significant downward trend was observed in the highest ICSEA quintile (Fig. 1b). For the moderate ICSEA groups (quintiles 2–4), prevalence of overweight and obesity was stable between 2009 and 2018.

**Fig. 2:**
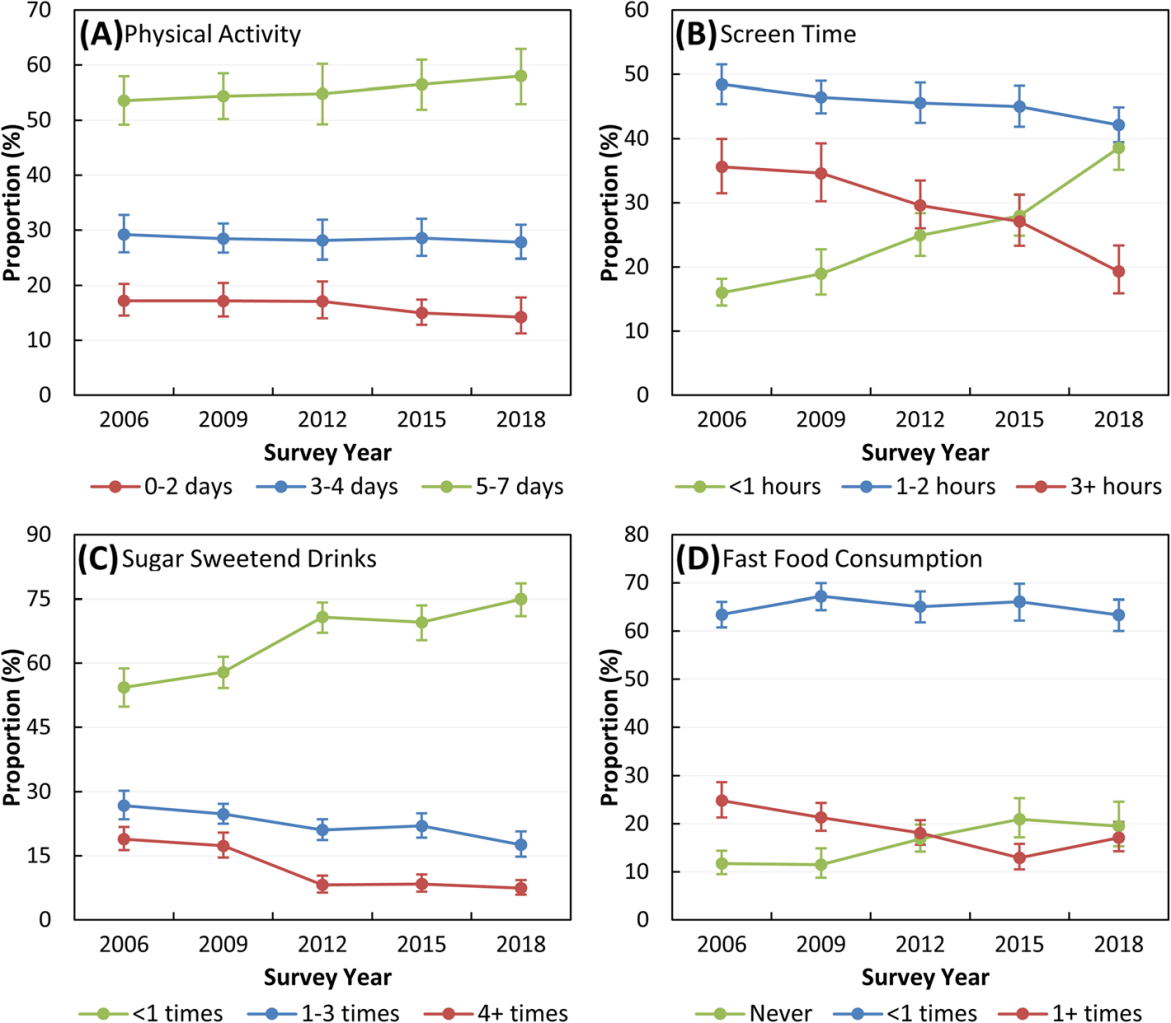
Prevalence of weight-related risk factors in ACT Year 6 children, 2006–2018. **a** Days per week of physical activity for at least 60 min per day; **b** Screen time per day on weekdays; **c** Frequency of sugar-sweetened drinks per week; and **d** Frequency of fast food consumption per week. Error bars indicate 95% confidence intervals for weighted proportions

There was a slight non-significant increase in the proportion of children reporting doing at least 60 min per day of physical activity for 5–7 days per week (Fig. 2a). The proportion of children spending less than 1 h per day watching TV and using a computer on weekdays also increased significantly, from 16.0% (95% CI: 14.0–18.2%) in 2006 to 38.5% (CI: 35.1–42.1%) in 2018 (Fig. 2b). There were significant increases, too, in the proportions of children reporting consuming less than one sugar-sweetened soft drink (SSD) per week (from 54.3% (CI: 49.8–58.8%) in 2006 to 75.0% (CI: 71.0–78.6%) in 2018, Fig. 2c) and never eating from a fast food outlet (from 11.7% (CI: 9.5–14.4%) in 2006 to 19.5% (CI: 15.3–24.6%) in 2018, Fig. 2d).

Levels of physical activity differed by sex, with 62.0% (95% CI: 59.2–64.8%) of boys reporting doing physical activity for 5–7 days per week, compared to 49.0% (95% CI: 46.3–51.6%) of girls. Boys (30.0%; CI: 28.0–32.1%) were more likely than girls (18.8%; CI: 17.3–20.4%) to meet the physical activity guideline of at least 60 min every day.

**Fig. 3:**
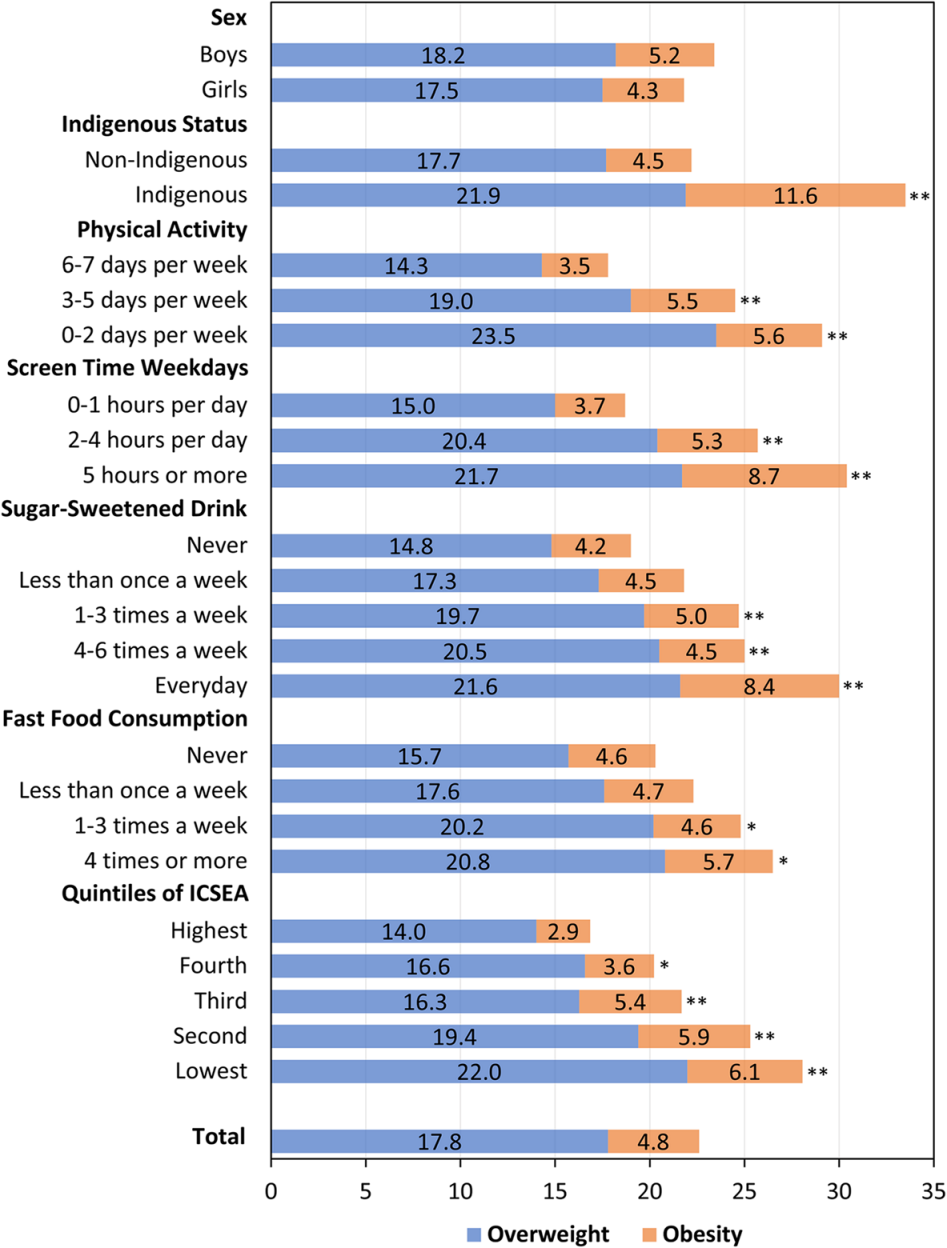
Prevalence (%) of overweight and obesity in ACT Year 6 children by selected demographic and behavioural factors, 2006–2018. Statistically significant differences comparing to the reference group (the first category) for the prevalence of overweight and obesity combined were indicated by * (*p* < 0.05) and ** (*p* < 0.01). ICSEA: Index of Community Socio-Educational Advantage

Figure 3 shows the prevalence of overweight and obesity by selected demographic and behavioural factors. Prevalence was significantly higher among Indigenous children, those in the lower ICSEA quintiles, and those reporting lower levels of physical activity, higher amounts of screen time and greater fast food and SSD consumption (Fig. 3). The unadjusted and adjusted relative risks for being overweight or obese associated with selected demographic and behavioural risk factors are presented in Table 2. Fast food and SSD consumption, physical activity, and sedentary behaviours were independently associated with overweight and obesity (Table 2). In the model adjusting for sex, Indigenous status, ICSEA quintiles and selected behavioural factors, higher screen time and lower physical activity were associated with a significantly increased risk of being overweight or obese (Table 2). However, after adjusting for other factors, the associations with SSD and fast food consumption were no longer significant, so they were not included in the final multiple regression model (Table 2). This change in statistical significance is likely due to the co-occurrence (correlation) of health-related risk factors in children.

**Table 2:** Unadjusted and adjusted prevalence ratios for overweight and obesity associated with selected demographic and behavioural factors in ACT Year 6 children, 2006–2018

Characteristics	Prevalence Ratios (95% CI)	
	Unadjusted	Adjusted^a^
Demographic factors		
Sex		
Boys	Ref.	Ref.
Girls	0.93 (0.85, 1.02)	0.90 (0.82, 0.98)
Indigenous status		
Non-Indigenous	Ref.	Ref.
Indigenous	1.51 (1.25, 1.83)	1.44 (1.19, 1.76)
ICSEA quintile (school-level)		
Highest (least deprived)	Ref.	Ref.
Fourth	1.20 (1.02, 1.41)	1.19 (0.97, 1.46)
Third	1.29 (1.09, 1.52)	1.27 (1.03, 1.55)
Second	1.50 (1.29, 1.75)	1.36 (1.12, 1.65)
Lowest (most deprived)	1.66 (1.43, 1.94)	1.52 (1.25, 1.84)
Behavioural factors		
Physical activity (60 m/d)		
6–7 days per week	Ref.	Ref.
3–5 days per week	1.37 (1.24, 1.53)	1.35 (1.21, 1.51)
0–2 days per week	1.64 (1.44, 1.86)	1.56 (1.37, 1.78)
TV and computer time weekdays		
0–1 h per day	Ref.	Ref.
2–4 h per day	1.38 (1.25, 1.52)	1.29 (1.17, 1.43)
5 h or more	1.63 (1.40, 1.89)	1.44 (1.23, 1.68)
Sugar-sweetened drink consumption^b^		
Never	Ref.	–
Less than once a week	1.15 (1.00, 1.33)	–
1–3 times a week	1.30 (1.11, 1.52)	–
4–6 times a week	1.32 (1.08, 1.61)	–
Everyday	1.58 (1.25, 2.00)	–
Fast food consumption^b^		
Never	Ref.	–
Less than once a week	1.10 (0.96, 1.26)	–
1–3 times a week	1.23 (1.04, 1.44)	–
4 times a week or more	1.31 (1.01, 1.71)	–

^a^Adjusted for sex, Indigenous status, ICSEA (Index of Community Socio-Educational Advantage), physical activity and screen time

^b^Stepwise regression method was used for selecting variables for inclusion in the multiple regression model. SSD and fast food consumption were not included in the final model because, after adjustment for other factors, the associations were no longer significant

**Fig. 4:**
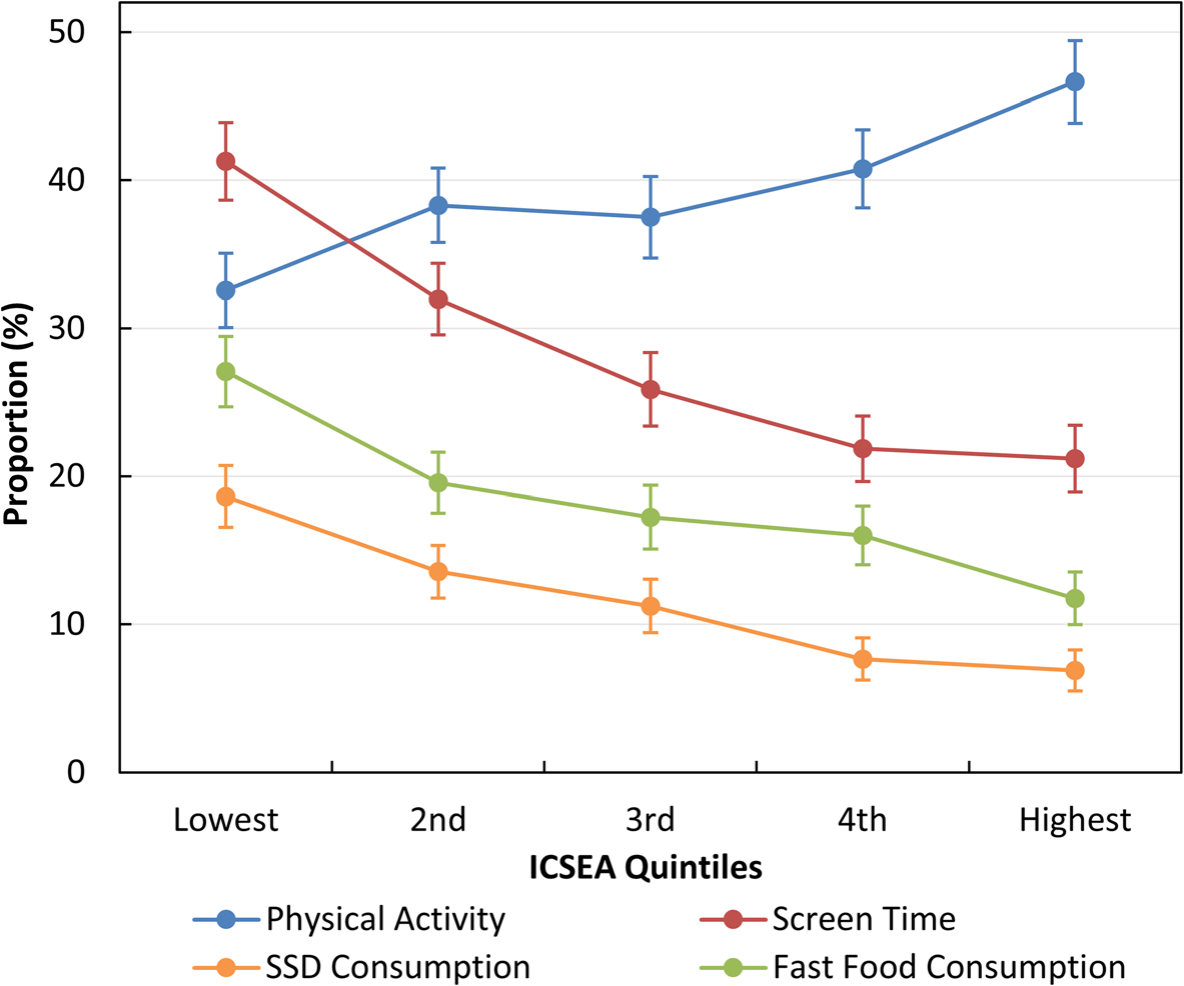
Association between ICSEA (Index of Community Socio-Educational Advantage) and the proportion of children reporting 6–7 days per week of physical activity for at least 60 min per day (blue circles and line), 3 or more hours per day of screen time on weekdays (red circles and line), 4 or more times per week of sugar-sweetened drinks consumption (orange circles and line), and more than once per week of fast food consumption (green circles and line). Error bars indicate 95% confidence intervals

SES gradient, as measured by school-level ICSEA, was highly associated with risk of being overweight or obese (Table 2, Fig. 3). Children in the lowest ICSEA quintile (most deprived) were more likely to be overweight and obese compared to those in the moderate and highest ICSEA quintiles (Table 2). Children in the lower ICSEA quintiles also reported lower physical activity, higher screen time and greater fast food and SSD consumption compared to those in higher ICSEA quintiles (Fig. 4).

There was large variance in the prevalence of overweight and obesity among schools. The multilevel regression analysis showed that the selected demographic and behavioural factors explained the majority (71.2%) of variance across schools.

## Discussion

Based on ACTPANS data for ACT Year 6 children, aged mainly 11–12 years, from 2006 to 2018, prevalence of overweight and obesity remained stable in girls but declined in boys. The reasons for the differences by sex in trends of overweight and obesity in this study are unclear. Boys and girls differ in body composition, patterns of growth, hormone biology, as well as susceptibility to certain social, cultural, and environmental factors [31, 32]. However, studies of gender differences in weight status and lifestyle behaviours at various childhood developmental stages are limited [31, 33, 34]. In the current study, levels of physical activity differed by gender, with a higher proportion of boys (30.0%) meeting the physical activity guidelines compared to girls (18.8%). Differences between boys and girls in levels of physical activity and other lifestyle choices have also been reported previously [33–35]. It has been suggested that gender differences should be considered in intervention programs designed to promote behavioural change in children [33, 36].

There is also evidence from other recent studies conducted in Australia and other developed countries of a plateau or, even, a decline in prevalence of overweight and obesity in children and adolescents [1, 19–23, 37]. It has been postulated that these trends reflect a positive effect of public health campaigns designed to prevent excess weight gain in children at the local, state and national level [38, 39]. Since 2012, a range of health promotion programs have been developed and implemented in the ACT to promote physical activity and healthy eating in school children [40]. For example, the *Ride or Walk to School* program was launched in 2012 to build capacity of schools to actively support and encourage students to ride or walk to school. Additional funding was committed to expand the program and, as of May 2019, 65% (84 of the 128) of primary and high schools in the ACT are participating. Further, the *ACT Public School Food and Drink Policy 2015* was implemented to prevent the sale of sugary drinks in ACT government schools [40]. Some non-government schools have also adopted this policy, although it is not mandated.

Analysis of the ACTPANS data indicated a reduction in unhealthy behaviours that contribute to overweight and obesity between 2006 and 2018. For example, concurrent with a slight increase in physical activity, screen time and frequency of fast food and SSD consumption decreased over the same period. Consistent with other studies [13, 41], our findings confirm that physical activity, screen time, and consumption of SSD and fast food are important predictors of childhood weight status. However, because health-related risk behaviours tend to co-occur in children and adolescents [13, 41], the relative importance of individual factors in the development of overweight and obesity is difficult to determine.

In the current study, socioeconomic status, based on school ICSEA, was significantly associated with excess weight in ACT Year 6 school children. The socioeconomic gradient in childhood overweight and obesity has been reported previously [14–18]. Of greater concern is the widening socioeconomic inequalities in recent years. Since 2006, the estimated prevalence of overweight and obesity has remained high in the lowest SES groups, but a concurrent downward trend was observed in the highest SES group, leading to increasing disparity between SES groups.

The effect of SES is generally understood to be mediated through differences in weight-related behaviours [17, 37, 42, 43]. Compared to those from high SES background, ACT Year 6 children of low SES background reported lower levels of physical activity, higher levels of screen time and greater fast food and SSD consumption. These differences may reflect difficulties experienced by lower socio-economic groups in receiving or actively responding to health promotion messages [23, 25, 37]. This lack of effectiveness in low SES groups, which has the unintended effect of widening inequality [23, 44], suggests that more targeted intervention strategies or regulations are needed. The context in which a child lives involves complex interactions among individual, family, school and community factors. Intervention studies suggest that effective healthy lifestyle promotion in children requires multiple setting approaches, combining education, environmental change and family involvement [15, 45–47]. Several studies highlight the importance of family influence in promoting healthy life behaviours in children and support the development of health promotion strategies and interventions involving families [43, 47–50].

In the current study, diet, physical activity and sedentary behaviours were assessed using self-reported questionnaires, which are prone to recall bias. School-level ICSEA was used to approximate student’s SES background because family SES measures, such as parents’ education, occupation and income, at the individual level were not collected in the ACTPANS data. ACT schools have higher ICSEA scores relative to the national average. The ICSEA values for sampled schools ranged from 954 to 1184 with an average of 1095, whereas the national average is 1000; thus, the socioeconomic effect may not be accurately represented in these results. Over time, there was a slight increase in the proportion of children from non-government schools in the sample; however, this has been adjusted for using post-stratification sampling weights. Due to the correlation among weight-related risk factors, the relative importance of each factor is also not well captured. Strengths of our study include the large representative sample, high response rate and measured, rather than self-reported, height and weight. The multilevel modelling method used in the study allows for the adjustment of factors at different levels that may confound the association between weight status and the selected exposures.

## Conclusion

Recent trends in childhood overweight and obesity in the ACT are promising and may reflect the positive effect of public health efforts to halt the epidemic rise in overweight and obesity in children. However, prevalence of overweight and obesity remains unacceptably high, especially in those from low socioeconomic backgrounds. Additional prevention efforts are required to achieve substantial improvement and address the socioeconomic disparity.

## Data Availability

De-identifiable data are available on request from ACT Health, but conditions apply.

## Abbreviations

ACT: Australian Capital Territory
ACTPANS: Australian Capital Territory physical activity and nutrition survey
CI: Confidence interval
ICSEA: Index of Community Socio-Educational Advantage
SES: Socioeconomic status
SSD: Sugar-sweetened drinks

## References

[1] NCD-RisC (2017). Worldwide trends in body-mass index, underweight, overweight, and obesity from 1975 to 2016: a pooled analysis of 2416 population-based measurement studies in 128.9 million children, adolescents, and adults. Lancet.

[2] Lobstein T, Baur L, Uauy R (2004). Obesity in children and young people: a crisis in public health. Obes Rev.

[3] Singh AS, Mulder C, Twisk JWR, Van Mechelen W, Chinapaw MJM (2008). Tracking of childhood overweight into adulthood: a systematic review of the literature. Obes Rev.

[4] Geserick M, Vogel M, Gausche R, Lipek T, Spielau U, Keller E (2018). Acceleration of BMI in early childhood and risk of sustained obesity. N Engl J Med.

[5] Park MH, Falconer C, Viner RM, Kinra S (2012). The impact of childhood obesity on morbidity and mortality in adulthood: a systematic review. Obes Rev.

[6] Freemark M, Freemark MS (2018). Childhood obesity in the modern age: global trends, determinants, complications, and costs. Pediatric obesity: etiology, pathogenesis and treatment.

[7] Reilly JJ, Kelly J (2011). Long-term impact of overweight and obesity in childhood and adolescence on morbidity and premature mortality in adulthood: systematic review. Int J Obes.

[8] Sonntag D, Ali S, De Bock F (2016). Lifetime indirect cost of childhood overweight and obesity: a decision analytic model. Obesity.

[9] Puhl RM, Latner JD (2007). Stigma, obesity, and the health of the nation's children. Psychol Bull.

[10] Quek Y-H, Tam WWS, Zhang MWB, Ho RCM (2017). Exploring the association between childhood and adolescent obesity and depression: a meta-analysis. Obes Rev.

[11] Ng M, Fleming T, Robinson M, Thomson B, Graetz N, Margono C (2014). Global, regional, and national prevalence of overweight and obesity in children and adults during 1980-2013: a systematic analysis for the global burden of disease study 2013. Lancet.

[12] Australian Bureau of Statistics (2018). National health survey, first results, 2017–18.

[13] Field AE, Goran MI (2017). Epidemiology of childhood obesity and associated risk factors: an overview. Childhood obesity: causes, consequences, and intervention approaches.

[14] Barriuso L, Miqueleiz E, Albaladejo R, Villanueva R, Santos JM, Regidor E (2015). Socioeconomic position and childhood-adolescent weight status in rich countries: a systematic review, 1990–2013. BMC Pediatr.

[15] Mech P, Hooley M, Skouteris H, Williams J (2016). Parent-related mechanisms underlying the social gradient of childhood overweight and obesity: a systematic review. Child Care Health Dev.

[16] Akkoyun-Farinez J, Omorou AY, Langlois J, Spitz E, Böhme P, Quinet M-H (2018). Measuring adolescents’ weight socioeconomic gradient using parental socioeconomic position. Eur J Pub Health.

[17] Chung A, Peeters A, Gearon E, Backholer K (2018). Contribution of discretionary food and drink consumption to socio-economic inequalities in children’s weight: prospective study of Australian children. Int J Epidemiol.

[18] Watts AW, Mason SM, Loth K, Larson N, Neumark-Sztainer D (2016). Socioeconomic differences in overweight and weight-related behaviors across adolescence and young adulthood: 10-year longitudinal findings from project EAT. Prev Med.

[19] Kern E, Chan NL, Fleming DW, Krieger JW (2014). Declines in student obesity prevalence associated with a prevention initiative - King County, Washington, 2012. Morb Mortal Wkly Rep.

[20] Ogden CL, Carroll MD, Lawman HG, Fryar CD, Kruszon-Moran D, Kit BK (2016). Trends in obesity prevalence among children and adolescents in the United States, 1988-1994 through 2013-2014. JAMA.

[21] Wen X, Gillman MW, Rifas-Shiman SL, Sherry B, Kleinman K, Taveras EM (2012). Decreasing prevalence of obesity among young children in Massachusetts from 2004 to 2008. Pediatrics.

[22] Rodd C, Sharma AK (2016). Recent trends in the prevalence of overweight and obesity among Canadian children. CMAJ.

[23] Hardy LL, Mihrshahi S, Gale J, Drayton BA, Bauman A, Mitchell J (2017). 30-year trends in overweight, obesity and waist-to-height ratio by socioeconomic status in Australian children, 1985 to 2015. Int J Obes.

[24] Chung A, Backholer K, Wong E, Palermo C, Keating C, Peeters A (2016). Trends in child and adolescent obesity prevalence in economically advanced countries according to socioeconomic position: a systematic review. Obes Rev.

[25] Stamatakis E, Wardle J, Cole TJ (2010). Childhood obesity and overweight prevalence trends in England: evidence for growing socioeconomic disparities. Int J Obes.

[26] Jansen PW, Mensah FK, Nicholson JM, Wake M (2013). Family and neighbourhood socioeconomic inequalities in childhood trajectories of BMI and overweight: longitudinal study of Australian children. PLoS One.

[27] Cole TJ, Lobstein T (2012). Extended international (IOTF) body mass index cut-offs for thinness, overweight and obesity. Pediatr Obes.

[28] National Health and Medical Research Council (2013). Australian dietary guidelines.

[29] ACARA (2015). Guide to understanding ICSEA (index of socio-educational advantage) values: from 2013 onwards.

[30] Norton EC, Miller MM, Kleinman LC (2013). Computing adjusted risk ratios and risk differences in Stata. Stata J.

[31] Wisniewski AB, Chernausek SD (2009). Gender in childhood obesity: family environment, hormones, and genes. Gend Med.

[32] Campbell MK (2016). Biological, environmental, and social influences on childhood obesity. Pediatr Res.

[33] Sweeting HN (2008). Gendered dimensions of obesity in childhood and adolescence. Nutr J.

[34] Santiago S, Zazpe I, Martí A, Cuervo M, Martínez JA (2013). Gender differences in lifestyle determinants of overweight prevalence in a sample of southern European children. Obes Res Clin Pract.

[35] Hobin EP, Leatherdale ST, Manske S, Dubin JA, Elliott S, Veugelers P (2012). A multilevel examination of gender differences in the association between features of the school environment and physical activity among a sample of grades 9 to 12 students in Ontario, Canada. BMC Public Health.

[36] Simen-Kapeu A, Veugelers PJ (2010). Should public health interventions aimed at reducing childhood overweight and obesity be gender-focused?. BMC Public Health.

[37] Sánchez-Cruz J-J, de Ruiter I, Jiménez-Moleón JJ, García L, Sánchez M-J (2018). Stabilization and reversal of child obesity in Andalusia using objective anthropometric measures by socioeconomic status. BMC Pediatr.

[38] Wabitsch M, Moss A, Kromeyer-Hauschild K (2014). Unexpected plateauing of childhood obesity rates in developed countries. BMC Med.

[39] Nobari TZ, Whaley SE, Prelip ML, Crespi CM, Wang MC (2018). Trends in socioeconomic disparities in obesity prevalence among low-income children aged 2–4 years in Los Angeles County, 2003–2014. Child Obes.

[40] ACT Government. Healthy Weight Initiative 2016–17 progress report. Canberra: ACT Government; 2017.

[41] Leech RM, McNaughton SA, Timperio A (2014). The clustering of diet, physical activity and sedentary behavior in children and adolescents: a review. Int J Behav Nutr Phys Act.

[42] Hanson MD, Chen E (2007). Socioeconomic status and health behaviors in adolescence: a review of the literature. J Behav Med.

[43] Mihrshahi S, Drayton BA, Bauman AE, Hardy LL (2018). Associations between childhood overweight, obesity, abdominal obesity and obesogenic behaviors and practices in Australian homes. BMC Public Health.

[44] Pescud M, Sargent G, Kelly P, Friel S (2019). How does whole of government action address inequities in obesity? A case study from Australia. Int J Equity Health.

[45] Eagle TF, Sheetz A, Gurm R, Woodward AC, Kline-Rogers E, Leibowitz R (2012). Understanding childhood obesity in America: linkages between household income, community resources, and children's behaviors. Am Heart J.

[46] van de Gaar VM, Jansen W, van Grieken A, Borsboom GJJM, Kremers S, Raat H (2014). Effects of an intervention aimed at reducing the intake of sugar-sweetened beverages in primary school children: a controlled trial. Int J Behav Nutr Phys Act.

[47] Langford R, Bonell C, Jones H, Campbell R (2015). Obesity prevention and the health promoting schools framework: essential components and barriers to success. Int J Behav Nutr Phys Act.

[48] Lindsay AC, Sussner KM, Kim J, Gortmaker S (2006). The role of parents in preventing childhood obesity. Futur Child.

[49] Vander Ploeg KA, Maximova K, Kuhle S, Simen-Kapeu A, Veugelers PJ (2012). The importance of parental beliefs and support for physical activity and body weights of children: a population-based analysis. Can J Public Health.

[50] Rhee KE, Boutelle KN, Goran MI (2017). Parent- and family-level factors associated with childhood obesity. Childhood obesity: causes, consequences, and intervention approaches.

